# Development and Evaluation of a Sensitive PCR-ELISA System for Detection of *Schistosoma* Infection in Feces

**DOI:** 10.1371/journal.pntd.0000664

**Published:** 2010-04-20

**Authors:** Luciana Inácia Gomes, Letícia Helena dos Santos Marques, Martin Johannes Enk, Maria Cláudia de Oliveira, Paulo Marcos Zech Coelho, Ana Rabello

**Affiliations:** 1 Laboratório de Pesquisas Clínicas, Centro de Pesquisas René Rachou, Fundação Oswaldo Cruz (Fiocruz), Belo Horizonte, Minas Gerais, Brazil; 2 Laboratório de Esquistossomose, Centro de Pesquisas René Rachou, Fundação Oswaldo Cruz (Fiocruz), Belo Horizonte, Minas Gerais, Brazil; Swiss Tropical Institute, Switzerland

## Abstract

**Background:**

A PCR-enzyme-linked immunosorbent assay (PCR-ELISA) was developed to overcome the need for sensitive techniques for the efficient diagnosis of *Schistosoma* infection in endemic settings with low parasitic burden.

**Methodology/Principal Findings:**

This system amplifies a 121-base pair tandem repeat DNA sequence, immobilizes the resultant 5′ biotinylated product on streptavidin-coated strip-well microplates and uses anti-fluorescein antibodies conjugated to horseradish peroxidase to detect the hybridized fluorescein-labeled oligonucleotide probe. The detection limit of the *Schistosoma* PCR-ELISA system was determined to be 1.3 fg of *S. mansoni* genomic DNA (less than the amount found in a single cell) and estimated to be 0.15 *S. mansoni* eggs per gram of feces (fractions of an egg). The system showed good precision and genus specificity since the DNA target was found in seven *Schistosoma* DNA samples: *S. mansoni*, *S. haematobium*, *S. bovis*, *S. intercalatum*, *S. japonicum*, *S. magrebowiei* and *S. rhodaini*. By evaluating 206 patients living in an endemic area in Brazil, the prevalence of *S. mansoni* infection was determined to be 18% by examining 12 Kato-Katz slides (41.7 mg/smear, 500 mg total) of a single fecal sample from each person, while the *Schistosoma* PCR-ELISA identified a 30% rate of infection using 500-mg of the same fecal sample. When considering the Kato-Katz method as the reference test, artificial sensitivity and specificity rates of the PCR-ELISA system were 97.4% and 85.1%, respectively. The potential for estimating parasitic load by DNA detection in feces was assessed by comparing absorbance values and eggs per gram of feces, with a Spearman correlation coefficient of 0.700 (*P*<0.0001).

**Conclusions/Significance:**

This study reports the development and field evaluation of a sensitive *Schistosoma* PCR-ELISA, a system that may serve as an alternative for diagnosing *Schistosoma* infection.

## Author Summary

Schistosomiasis is a neglected disease caused by worms of the genus *Schistosoma*. The transmission cycle requires contamination of bodies of water by parasite eggs present in excreta, specific snails as intermediate hosts and human contact with water. Fortunately, relatively safe and easily administrable drugs are available and, as the outcome of repeated treatment, a reduction of severe clinical forms and a decrease in the number of infected persons has been reported in endemic areas. The routine method for diagnosis is the microscopic examination but it fails when there are few eggs in the feces, as usually occurs in treated but noncured persons or in areas with low levels of transmission. This study reports the development of the PCR-ELISA system for the detection of *Schistosoma* DNA in human feces as an alternative approach to diagnose light infections. The system permits the enzymatic amplification of a specific region of the DNA from minute amounts of parasite material. Using the proposed PCR-ELISA approach for the diagnosis of a population in an endemic area in Brazil, 30% were found to be infected, as compared with the 18% found by microscopic fecal examination. Although the technique requires a complex laboratory infrastructure and specific funding it may be used by control programs targeting the elimination of schistosomiasis.

## Introduction

Schistosomiasis affects 200 million people and about 779 million people live in endemic areas in the Middle East, South America, Caribbean, Southeast Asia and particularly sub-Saharan Africa [1]. Population- and treatment-based control programs have been successful in reducing the intensity of infection and severe morbidities associated with schistosomiasis; however, transmission remains active in highly endemic areas, and recurring low-level reinfection is likely to be associated with subtle but persistent morbidities such as anemia, malnutrition and diminished performance status [2]–[4]. In the presence of these conditions, the assessment of infection becomes less reliable since the currently used diagnostic methods are not sufficiently sensitive to accurately determine the prevalence of schistosomiasis or parasite burden in order to eventually achieve elimination of the disease [5], [6].

Microscopic demonstration of the parasite's eggs in feces or urine remains the most wide-spread tool for schistosomiasis diagnosis. The Kato-Katz technique [7] is currently the most used method for fecal examination because it is quantitative, relatively inexpensive and simple. A significant increase in the sensitivity of the method is gained by microscopic examination of multiple samples [8], [9], but this is a limiting procedure for field work.

To overcome the current limitations with respect to diagnosis, the simultaneous use of different diagnostic methods, such as antibody detection followed by stool examination of seropositive individuals, has been applied to monitor the human population and to identify the small number of infected people once morbidity control is achieved [6]. However, because antibody detection methods often cannot distinguish between current and past infection and may also present a high level of crossreactivity, molecular tools should be considered despite their higher cost and the requirement for special laboratory equipment [10].

Hamburger et al. [11] described a 121-base pair tandem repeat DNA sequence present in 12% of *Schistosoma mansoni* genome. This sequence has been successfully used in PCR-based approaches for the detection of the parasite in snails [12], monitoring of cercariae in water bodies [13] and diagnosis of human infection using fecal or serum samples [14] and, more recently, plasma samples [15]. In a population study, the prevalence of *S. mansoni* infection was determined to be 31% when three fecal samples were examined using the Kato-Katz technique, but the prevalence rose to 38% when the PCR technique developed by Pontes et al. [14] was employed using only one fecal sample [16]. The same result was observed by another group in a recent study assessing the marginal error of Kato-Katz examinations for diagnosis and cure evaluation of *S. mansoni* infection in areas of low endemicity [17].

Conventional PCR requires several steps after DNA amplification, including electrophoresis or blotting and hybridization, which are limited in the number of samples that can be conveniently analyzed. The PCR-enzyme-linked immunosorbent assay (PCR-ELISA) consists of an alternative process for large-scale screening that allows for semi-quantitative analysis. This technique combines an immunological method to quantify the PCR product directly after immobilization of biotinylated DNA on a microplate [18], [19]. The advantage of PCR-ELISA as compared to PCR-electrophoresis is that it makes use of standard equipment widely used for the processing of ELISAs, and the reagents used are easy to obtain commercially. Therefore, PCR-ELISA allows for the use of PCR-based DNA diagnosis for routine purposes in laboratories in less developed countries with fewer resources.

The aim of this work was to design a *Schistosoma* PCR-ELISA system as an improvement over the PCR assay previously developed by our group to detect *S. mansoni* DNA in human fecal samples [14]. The performance of the new assay was evaluated with fecal specimens from a Brazilian endemic area for *S. mansoni* infection and compared with the parasitological Kato-Katz technique for detection and estimation of the intensity of infection.

## Materials and Methods

### *S. mansoni* eggs and artificial egg-spiked fecal samples

For use throughout the development of the *Schistosoma* PCR-ELISA system and for the estimation of its lower detection limit, *S. mansoni* eggs were obtained from the livers of Swiss albino mice 60 days after infection with 150 cercariae and stored at −20°C in 1.7% saline until use [20]. The animals were handled according to local and federal regulations, and the research protocol was approved by the Fiocruz Committee on Animal Research (License L-0118/09).

The number of eggs in saline suspension was quantified using a Neubauer chamber, and a solution containing approximately 2,000 eggs was used for DNA extraction. A negative fecal sample (verified to be free of *S. mansoni* eggs by the Kato-Katz technique and negative for the *Schistosoma* DNA detection by the PCR-ELISA system) was spiked with the egg-saline solution. The egg count was assessed by the Kato-Katz method, and approximately 500 mg of screened feces were used for DNA extraction.

### DNA from other *Schistosoma* species

Samples of genomic DNA from the human parasites *S. haematobium*, *S. intercalatum* and *S. japonicum*, and also from *S. bovis*, *S. magrebowiei* and *S. rhodaini* were provided by the Laboratório de Parasitologia Celular e Molecular (Centro de Pesquisas René Rachou, Fiocruz, Brazil) and used to assess the genus specificity of the *Schistosoma* PCR-ELISA system.

### Study population

Two hundred and six people from Pedra Preta, Minas Gerais, Brazil, an endemic area for *S. mansoni* infection, participated in this study. The group was composed of 69 children (female/male: 33/36; age range of 1–17 years) and 137 adults (female/male: 66/71; age range of 18–86 years). Thirty-six healthy members of the laboratory staff participated as negative controls throughout the development of the assay. Written informed consent was obtained from all adult participants and from parents or legal guardians of minors. This study was approved by the Ethics Committee of the Centro de Pesquisas René Rachou, Fiocruz, Brazil (No. 14/2008).

For PCR-ELISA, fecal samples were collected and stored at −70°C until DNA extraction. All samples were evaluated for the presence of *S. mansoni* eggs by the Kato-Katz method. Twelve glass slides (41.7 mg/smear) of a single fecal sample were examined, resulting in a total sample weight of about 500 mg. Egg counts were expressed in eggs per gram of feces (epg), using the arithmetic mean of eggs counts obtained from the 12 slides multiplied by 24 [7]. The intensity of infection was calculated as the geometric mean of the individual egg counts.

Individuals with positive fecal examination results were treated with a single oral dose of praziquantel (50 or 60 mg/kg for adults and children, respectively), according to the recommendation of the Brazilian Ministry of Health.

### DNA extraction

Total DNA from 500 mg of each fecal sample and the DNA from a saline solution containing approximately 2,000 *S. mansoni* eggs were extracted using the QIAamp DNA Stool Mini Kit (Qiagen GmbH, Hilden, Germany), according to the manufacturer's recommendations and following the protocols “DNA Isolation from Large Amounts of Stool” and “Isolation of DNA from Stool for Pathogen Detection”. The heating step was performed at 95°C for 20 min to guarantee egg rupture. The concentration and purity of the DNA were determined spectrophotometrically by readings of *A_260_* and *A*_280_ (Eppendorf, Hamburg, Germany).

### Primers and probes

The *Schistosoma* PCR-ELISA system consisted of a biotin 5′-labeled forward primer (5′-GATCTGAATCCGACCAACCG-3′), an unlabeled reverse primer(5′-ATATTAACGCCCACGCTCTC-3′) and the fluorescein 5′-labeled probe (5′-TGGTTTCGGAGATACAACGA-3′). The primers used were previously described [14] and designed to amplify a 121-bp tandem repeat DNA sequence (GenBank accession number M61098) found in the genome of *S. mansoni* [11].

To control for variation in the efficiency of DNA extraction and PCR-amplification, all clinical samples were evaluated with the human beta actin PCR-ELISA system. The primers used were Aco1 (5′-ACCTCATGAAGATCCTCACC-3′) and Aco2 (5′-CCATCTCTTGCTCGAAGTCC-3′), which were previously described to target the fourth exon of the human beta actin gene (*ACTB*) [21]. In this assay, the sense primer (Aco1) was biotinylated at the 5′ end, and a fluorescein 5′-labeled probe (5′-TCTCCTTAATGCACGCACG-3′) was designed together with the *Schistosoma* probe described above using the program Primer3-web 0.4.0 [22] and submitted to homology searches on the National Center for Biotechnology Information website with nucleotide BLAST program using database Nucleotide collection (nr/nt) and Megablast option. Amplification primers, biotinylated primers and probes were purchased from Integrated DNA Technologies, Inc. (Coralville, Iowa, USA).

### PCR conditions

For amplification, fecal DNA samples were diluted fivefold, and 2 µl were used as the template. The same volume was used to amplify *S. mansoni* egg-derived DNA, artificial *S. mansoni* egg-spiked fecal DNA and DNA from other *Schistosoma* species. PCR was carried out in a final volume of 20 µl containing 2 µl of GeneAmp 10X PCR Gold Buffer (150 mM Tris-HCl, pH 8.0, 500 mM KCl), 2.0 U of Amplitaq Gold (Applied Biosystems, Foster City, CA, USA), 0.1 µg/µl of BSA (Sigma, St. Louis, MO, USA), 0.5 µM of each primer, 1.5 mM MgCl_2_ and 200 µM of each deoxynucleoside triphosphate (Promega, Madison, WI, USA). The cycling programs, preceded by 12 min at 95°C to activate the HotStart *Taq* polymerase, consisted of 15 cycles of 95°C for 1 min, 63°C for 1 min and 72°C for 30 s; 12 cycles of 80°C for 1 min, 63°C for 1 min and 72°C for 30 s and 7 cycles of 80°C for 1 min, 65°C for 1 min and 72°C for 30 s, followed by a final elongation step at 72°C for 7 min. Positive controls based on DNA from *S. mansoni* eggs were included in all tests. Negative controls containing all of the elements of the reaction mixture except DNA were also included in each PCR assay as surveillance for contamination.

A 120-bp segment of the *ACTB* gene was amplified in a separate tube containing fecal DNA, following the amplification protocol described above, except that the MgCl_2_ concentration used was 2 mM. The cycling program, preceded by 12 min at 95°C, consisted of 35 cycles of 95°C for 20 s, 60°C for 30 s and 72°C for 1 min, followed by a final elongation step at 72°C for 7 min.

The chance of PCR contamination was minimized by physical separation of the starting materials and the amplified products in different rooms; the rooms contained laminar flux chambers with UV light, and sterile, disposable laboratory supplies were used.

### Detection of PCR products

#### (i) Electrophoresis

Five microliters of PCR-amplified egg-derived DNA, artificial egg-spiked fecal DNA and DNA from other *Schistosoma* species were subjected to electrophoresis on 6% polyacrylamide gels and analyzed by silver staining.

#### (ii) ELISA

For the *Schistosoma* PCR-ELISA system, the detection of the biotin-labeled amplified DNA was carried out using the PCR Plate Detection Kit (Sigma, St. Louis, MO, USA), according to the manufacturer's recommendations. Briefly, samples (5 µl of PCR product mixed with 95 µl of dilution buffer) were added in triplicate to streptavidin-coated wells, and the plate was incubated at 37°C for 30 min. Denaturation solution was added to a final volume of 100 µl and incubated for 10 min at room temperature. After four washes with PBS-Tween 20 washing solution (PBS-T), 200 µl of PlateHyb hybridization buffer containing 0.2 pmol of fluorescein 5′-labeled probe and 20% of formamide was added to each well for incubation at 37°C for 45 min. The plate was then washed five times with PBS-T solution. Anti-fluorescein-horseradish peroxidase (HRP) conjugated antibody, diluted 500-fold in PBS-T solution, was added to each well in a final volume of 150 µl, and then the plate was incubated at 37°C for 45 min. The plate was again washed five times with PBS-T solution. TMB Liquid Substrate System was added to each well to a final volume of 100 µl, and the plate was incubated at room temperature for 5 min, kept in the dark during color development. Finally, 50 µl of stop solution provided by the commercial kit was added, and absorbance at 450 nm was determined in an ELISA reader (Bio-Rad, Hercules, CA, USA). Each PCR-ELISA reaction was performed with positive (DNA extracted from *S. mansoni* eggs) and negative controls (water and PCR negative control). All experiments were performed in triplicate, and data represent mean values. The cut-off value was calculated as the mean absorbance value of the negative controls (20 healthy persons from the laboratory staff) plus three standard deviations, as obtained with a single PCR-ELISA test [23]. As the mean absorbance value and standard deviation were 0.080±0.012, a sample was considered positive when the absorbance of the three measurements was greater than 0.116 (the cut-off value).

The *ACTB* PCR-ELISA system followed the same conditions described above, except that 10 µl of PCR product was used; the hybridization protocol was performed at 50°C without the addition of formamide, and the colorimetric substrate TMB was reacted for 30 min at room temperature in the dark. The cut-off value obtained for each PCR-ELISA test was calculated as twice the mean absorbance value of the PCR negative control [23].

### Statistical analysis

Data were processed with SPSS statistical software package 13.0 for Windows (SPSS Inc., Chicago, IL, USA) and GraphPad Prism 3.0.3 software (San Diego, CA, USA). All quantitative variables were individually assessed with the one-sample Kolmogorov-Smirnov test for normality. Absorbance readings and arithmetical means of the number of eggs per gram of feces and template DNA concentration (both transformed into log scale) were analyzed by Pearson's parametric correlation coefficient or Spearman's nonparametric correlation coefficient. In order to determine the variability of the assays, intra-assay (repeatability) and inter-assay (reproducibility) precision levels were measured by comparing the means ± the S.D. and reported as coefficients of variation (CVs, S.D./mean X 100%). Sensitivity, specificity and 95% confidence intervals (CIs) were calculated using the OpenEpi Version 2.3 program [24]. Agreement beyond chance was assessed using the Kappa index and interpreted according to Landis and Koch [25]: 1.00-0.81 is excellent, 0.80-0.61 is good, 0.60-0.41 is moderate, 0.40-0.21 is weak and 0.20-0.0 is negligible. The *X^2^* test was employed for the comparison of proportions. The level of significance was set at *P<*0.05.

## Results

### *Schistosoma* PCR-ELISA system development

**Figure 1:**
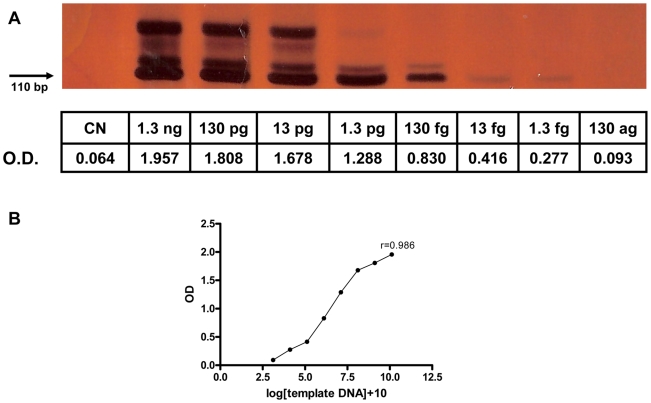
Analytical sensitivity of PCR-ELISA system. **A.** Analytical sensitivity of PCR followed by 6% polyacrylamide gel electrophoresis with silver staining and ELISA (optical density, OD) for 10-fold serial dilutions of genomic DNA extracted from a saline solution containing ∼2,000 *S. mansoni* eggs. The detection limit was 1.3 fg of genomic DNA. A ladder-type banding pattern was exhibited, as expected, due the amplification of the *Schistosoma* tandem-repeated unit, with the main DNA band of 110 bp present in all samples. **B.** The Pearson's correlation coefficient between PCR-ELISA OD and log[template DNA]+10 was 0.986 (*P*<0.0001).

The system's analytical sensitivity was evaluated using eight 10-fold serial dilutions ranging from 1.3 ng to 130 ag of genomic DNA extracted from a saline solution containing *S. mansoni* eggs. The limit of detection was 1.3 fg of genomic DNA, equal to that obtained by 6% polyacrylamide gel electrophoresis and silver staining, since the absorbance value for the sample with 130 ag of DNA was equivalent to the PCR negative control (0.093 and 0.064, respectively) (Figure 1A). The correlation between the numeric results (optical density readings) of the PCR-ELISA and the log number of the template DNA was significant, with a Pearson's correlation coefficient of 0.986 (*P*<0.0001) (Figure 1B). The analytical sensitivity of the assay was also evaluated with 10-fold dilutions of DNA extracted from a negative fecal sample spiked with an *S. mansoni* egg-saline solution determined by the Kato-Katz method to contain 1,534 epg. The *Schistosoma* PCR-ELISA system was consistently able to detect a sample estimated to contain 0.1534 epg.

**Figure 2:**
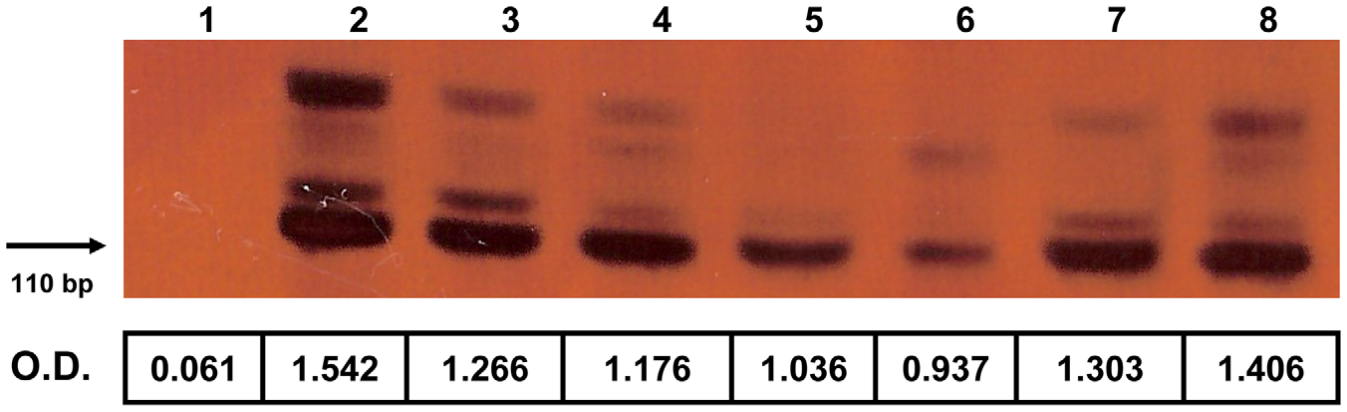
Analytical specificity of PCR followed by 6% polyacrylamide gel electrophoresis with silver staining and ELISA (optical density, OD) for purified DNA from adult *S. magrebowiei* (lane 2), *S. rhodaini* (lane 3), *S. japonicum* (lane 4), *S. intercalatum* (lane 5), *S. haematobium* (lane 6), *S. bovis* (lane 7) and *S. mansoni* (lane 8) worms. Lane 1, PCR negative control. The genus specificity of the *Schistosoma* PCR-ELISA system was demonstrated by the amplification of the 121-bp tandem repeat DNA sequence in all samples with the same set of primers. Absorbance readings were also consistent with the positive control (*S. mansoni* DNA).

The genus specificity of PCR-ELISA was assessed with purified DNA from *S. mansoni*, *S. haematobium*, *S. bovis*, *S. intercalatum*, *S. japonicum*, *S. magrebowiei* and *S. rhodaini* adult worms. Results (Figure 2) showed a ladder of PCR products due the amplification of the *Schistosoma* tandem-repeated unit, with the main DNA band of 110 bp present in all samples, as expected, and absorbance readings comparable to the positive control (*S. mansoni* DNA).

To analyze the repeatability of the *Schistosoma* PCR-ELISA system, three positive and three negative fecal samples from patients were assessed in four replicates in a single run. The intra-assay CVs for absorbance values for the negative samples were: 5.6%, 7.4% and 8.3%; the CVs for the positive samples were 1.9%, 3.6% and 4.2%. In addition, to measure the reproducibility, four replicates of the same samples were assessed on different days in four different assays. The inter-assay variations of absorbance values for the positive DNA samples were 3.8%, 7.7% and 15.9%; the variations for the negative DNA samples were 14.2%, 15.4% and 18.7%.

### Analysis of patient samples

Fecal samples from 206 patients from an area in Brazil endemic for *S. mansoni* were analyzed by the *Schistosoma* PCR-ELISA system and also by the parasitological Kato-Katz technique. Comparison of the results obtained by the *Schistosoma* PCR-ELISA system and the Kato-Katz technique, based on examination of twelve slides (a total of 500 mg feces), is shown in Table 1. The geometric mean of the number of eggs per gram of feces estimated by the Kato-Katz technique for the positive samples was 18, which indicates a low intensity of infection [26]. The prevalence observed using the PCR-ELISA system (30%) was higher than that determined with the examination of twelve slides by the Kato-Katz technique (18%) (*X^2^* = 8.81, *P*<0.003). The Kappa index of 0.663 indicates good agreement between the two methods. Analysis of discordant results showed that 25 samples were positive only by the *Schistosoma* PCR-ELISA system, and one sample was positive only by the Kato-Katz technique. This patient had very low egg output (8 epg).

**Table 1:** Comparative evaluation of the *Schistosoma* PCR-ELISA system and the Kato-Katz technique for the diagnosis of *S. mansoni* infection.

	Positive	Negative	Total
**Kato-Katz fecal examination (2 slides – 83 mg feces)**			
PCR-ELISA			
Positive	26	36	62
Negative	1	143	144
Total	27	179	206
	Kappa index = 0.491		
**Kato-Katz fecal examination (12 slides – 500 mg feces)**			
PCR-ELISA			
Positive	37	25	62
Negative	1	143	144
Total	38	168	206
	Kappa index = 0.663		

Table 1 also shows a comparison of the results obtained by the *Schistosoma* PCR-ELISA system and the Kato-Katz technique, based on examination of two slides (a total of 83.4 mg feces), as is routinely done in epidemiological surveys. The Kappa index of 0.491 indicates a moderate agreement between the two methods. Analysis of discordant results showed that 36 samples were positive only by the *Schistosoma* PCR-ELISA system.

Diagnostic parameters were calculated by two different approaches: 1) taking two-slide Kato-Katz examination as the reference method for comparison or 2) considering twelve-slide Kato-Katz examination as the reference method. The sensitivity values of the *Schistosoma* PCR-ELISA system were high, regardless of the reference considered: 96.3% (95% CI, 81.7–99.3) for approach 1 and 97.4% (95% CI 86.5–99.5) for approach 2. Specificity values changed significantly depending on the reference used, being 79.9% (95% CI 73.4–85.1) for approach 1 and 85.1% (95% CI 78.7–89.7) for approach 2.

All 206 fecal samples analyzed by the *ACTB* PCR-ELISA system showed a positive result, ensuring that negative results correspond to true negative samples for the *Schistosoma* PCR-ELISA system rather than to a problem with sample degradation or PCR inhibition. Also, the remaining sixteen fecal samples of non-infected persons were evaluated to be positive for the *ACTB* PCR-ELISA system and negative for the *Schistosoma* PCR-ELISA system.

### Potential for semi-quantitative use of the PCR-ELISA system

**Figure 3:**
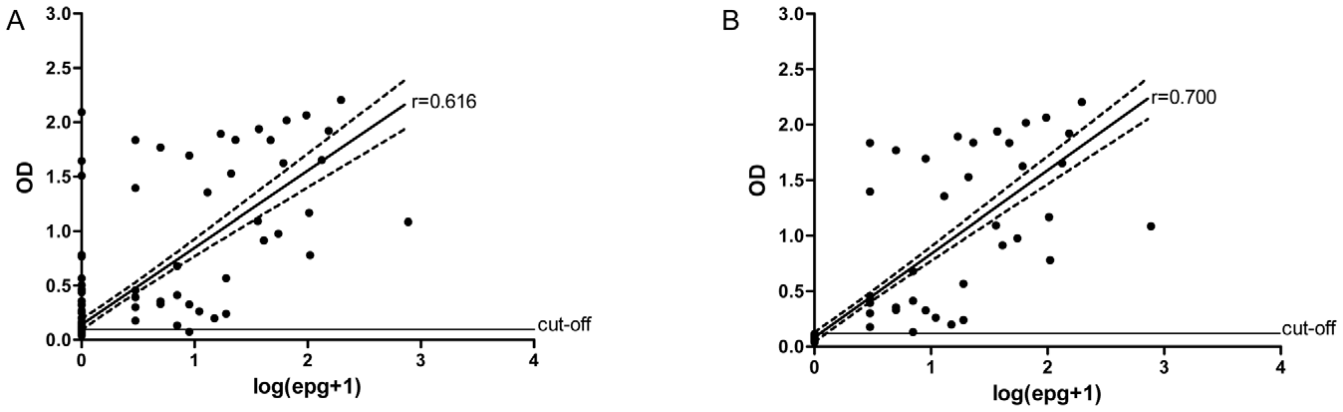
Correlation between log-transformed individual measurements of eggs per gram (epg) of feces and absorbance readings. **A.** A Spearman's correlation coefficient of 0.616 (*P*<0.0001) was found for a comparison considering all 206 samples. **B.** Considering positive and negative samples for the Kato-Katz technique and the *Schistosoma* PCR-ELISA system, a Spearman's correlation coefficient of 0.700 (*P*<0.0001) was observed. The continuous line represents the linear regression, and the hatched line represents the 95% CI.

The ability of the *Schistosoma* PCR-ELISA system to estimate the parasitic load was assessed, and a Spearman's correlation coefficient of 0.616 (*P*<0.0001) was found when comparing values of absorbance readings and epg (transformed into log[epg+1]) to those determined by the Kato-Katz technique with 12 examined slides (Figure 3A). When the correspondence between the methods was evaluated, considering only positive and negative samples for both, the correlation coefficient observed was 0.700 (*P*<0.0001) (Figure 3B).

Results obtained with the *Schistosoma* PCR-ELISA system based on two levels of intensity of infection (1–100 epg and >100 epg), according to the Kato-Katz stool examination method, are shown in Table 2. The high sensitivity of the *Schistosoma* PCR-ELISA system was evidenced by the high number of positive samples in both groups. The assay also revealed the potential to be considered semi-quantitative, as the mean absorbance readings corresponded to the intensity of infection.

**Table 2:** Results obtained with the *Schistosoma* PCR-ELISA system grouped into two levels of intensity of infection.

		*Schistosoma* PCR-ELISA system			
Intensity of infection^a^	Number	Negative	Positive	Positivity rate (%)	Mean OD^b^
Negative	168	143	25	14.9	0.069
1–100 epg	32	1	31	96.9	1.050
101+ epg	6	0	6	100	1.468

a Intensity of infection was based on examination of one fecal sample by the Kato-Katz technique (12 slides - 500 mg feces).

b Considering only concordant results between the Kato-Katz technique and the *Schistosoma* PCR-ELISA system; OD  =  optical density.

## Discussion

The control of schistosomiasis-related morbidity has become feasible due to the development of single-dose oral drugs such as oxamniquine and praziquantel, which are given to heavily infected patients (high worm burden) that were easily detected by field-applicable parasitological methods. With the transition to lower morbidity, there is need for more sensitive diagnostic methods. In an attempt to surpass this diagnostic limitation, a PCR-ELISA system was developed and evaluated as a new molecular assay for the diagnosis of *Schistosoma* infection. This system showed sound features, such as a low analytical limit of detection, genus specificity (and absence of cross-reactivity with other related parasites), good precision, high sensitivity, good specificity and also the potential for semi-quantitative analysis of parasitic load.

Evaluation of the analytical sensitivity of the *Schistosoma* PCR-ELISA system showed that it could accurately detect 1.3 fg of *S. mansoni* genomic DNA, which is equivalent to less than the DNA found in a single cell of this multicellular parasite, since its genome contains around 580 fg [27]. The high sensitivity of the assay is attainable due to the high copy number of the target sequence, which comprises approximately 12% of the *S. mansoni* genome [11]. In another approach, the analytical sensitivity was evaluated as the capability to detect DNA samples according to the egg count. The result obtained (0.1534 epg) corresponds to fractions of an egg.

The *Schistosoma* PCR-ELISA system was developed using primers designed by Pontes et al. [14], who demonstrated specificity by the absence of amplification when DNA from four related helminths (*Ascaris lumbricoides*, *Ancylostoma duodenale*, *Taenia solium* and *Trichiuris trichiura*) was used as templates. Further assay also showed no crossreactivity with *Fasciola hepatica* (data not shown). Additionally, the potential use of the previously described primers and probe and the current PCR protocol to detect genomic DNA from other *Schistosoma* species was assessed by the amplification of DNA extracted from worms of seven *Schistosoma* species (*S. mansoni*, *S. haematobium*, *S. bovis*, *S. intercalatum*, *S. japonicum*, *S. magrebowiei* and *S. rhodaini*). The results obtained confirmed what Hamburger et al. [11] partially addressed using a different set of primers that targets the internal part of the same DNA sequence: namely, this 121-bp tandem repeat is genus specific.

The precision of the *Schistosoma* PCR-ELISA system can be considered good, as the intra-assay variation was lower than 9%, and the inter-assay variation was around 3% to 19%. These percentages are similar to, or lower than, those calculated for other PCR-ELISA assay based on similar principles [28].

The choice of a reference test is crucial for the evaluation of any new testing method. The Kato-Katz technique is considered the test of choice to diagnose schistosomiasis in fecal samples and was chosen as a reference test for comparison with the *Schistosoma* PCR-ELISA system. Initially, for a traditional approach (approach 1 in this study), the comparison was made using two Kato-Katz slides, corresponding to 83.4 mg feces. The PCR-ELISA system was able to detect *S. mansoni* DNA in 36 fecal samples from patients with negative results by the Kato-Katz technique (two slides examined), demonstrating high sensitivity (96.3%), though lower specificity (79.9%). This specificity is likely to be artificial and incorrect, resulting from the less sensitive and inadequate reference method. In another approach (approach 2), twelve Kato-Katz slides were evaluated, corresponding to 500 mg of feces, the same quantity used to extract total DNA for analysis with the *Schistosoma* PCR-ELISA system. Again, the PCR-ELISA system detected more cases of infection with *S. mansoni*. Thus the sensitivity was high (97.4%), and the specificity was more satisfactory (85.1%). It is well documented that the Kato-Katz method lacks sensitivity if only a single fecal sample is examined, particularly in areas with high proportions of light-intensity infections. A small number of eggs unequally excreted over several days or patchily distributed may not be detected in the small amount of feces examined, negatively impacting on the method's sensitivity. In order to overcome these shortcomings, examination of an increased number of fecal samples as well as in the number of slides per specimen is required. There exists a general consensus that two Kato-Katz slides for each of three fecal samples yield enough sensitivity to obtain reliable data [29]-[32]. As this present study is part of a wider study in the endemic area of Pedra Petra, Minas Gerais, Brazil, three additional samples were collected on different days and analyzed by two Kato-Katz slides each (data not shown). From samples with discordant results between Kato-Katz analysis (twelve slides examined) and the *Schistosoma* PCR-ELISA, 12 out of 25 (48%) were positive in subsequent Kato-Katz examinations of additional samples. Therefore, a better explanation for these discordant results is that the *Schistosoma* PCR-ELISA system is more sensitive than the Kato-Katz technique considering the same amount of stool examined. That is, these cases correspond to *S. mansoni-*infected samples and not false-positive results. Although carryover contamination is often difficult to exclude as a cause of false-positive results in PCR-based diagnostic assays, the strict measures taken to avoid it throughout the entire handling and processing steps of the assay make the chances low.

Following both approaches, one sample was positive for the Kato-Katz technique and negative for the *Schistosoma* PCR-ELISA system. Since the parasitological method has an assumed specificity of 100%, this result can be most reasonably attributed to misdiagnosis by the PCR-ELISA. Inhibition of the amplification reaction by fecal compounds and/or DNA degradation during transportation of the sample from the field were considered the most likely technical causes. However, they were subsequently ruled out, as a positive result was obtained with the control *ACTB* PCR-ELISA system. Therefore, a better explanation seems to be the uneven distribution of a small quantity of eggs in the feces [33], since the sample had a very low parasite load (8 epg).

ELISA methods for the detection of PCR products provide an alternative to gel-based detection. Among other features, ELISAs prevent possible subjective interpretations of PCR results due to “nonspecific products” or “bands of unknown origin.” The main advantage of the procedure is the ability to process many samples in parallel (e.g., the 96-well microplate format), using instrumentation developed for processing ELISA for antibody detection. In addition, the procedure takes less than 2.5 hours to be performed (with the PCR Plate Detection Kit; Sigma).

The detection format of the commercial kit used here combines methodological features of PCR-ELISA methods described by Landgraf et al. [18] and Luneberg et al. [19]. Biotin-streptavidin binding was used for immobilization of amplification products on microtiter plates, taking advantage of the biotin moiety conjugated to one PCR primer. Subsequent hybridization was carried out with a fluorescein-labeled oligonucleotide probe, with the advantage of confirming the specificity of the test and allowing for detection by an anti-fluorescein-HRP conjugate.

Regardless of the fact that real-time PCR provides the best means to quantify PCR products, PCR-ELISA also allows for quantification, although it is usually considered a semi-quantitative technique, as it analyzes end-point amplification products. At the end-point, net synthesis is significantly reduced, inhibitory effects have accumulated and differences in initial starting template concentrations are masked [34]. These problems may be overcome by stopping the reaction in the exponential phase (as was done in the present study) or by requiring competitive amplification with very similar template DNA and primer pairs [18].

One classification used for epidemiological estimates of the intensity of infection of schistosomiasis on a community level considers the following groups: i) light, ≤100 eggs/gram of feces; ii) moderate, 101 to 400 eggs/gram of feces and iii) heavy, >400 eggs/gram of feces [26]. Even though the PCR-ELISA system developed in this study showed a satisfactory correlation coefficient between absorbance readings and egg counts per gram of feces, it seemed to be adequate to classify the samples according to the same parameters. However, as the performance of this new assay was evaluated in a low endemic setting, the number of samples with high egg counts was small, limiting statistical analysis. Thus, a statistically significant difference between mean absorbance values was observed only between the positive and negative groups (*P*<0.0001 for all). Nevertheless, an increase in the mean absorbance readings with increasing intensity of infection could be observed. Additional studies in areas of higher prevalence, resulting in a statistically significant number of patients with high egg counts per gram of feces, need to be carried out in order to validate the *Schistosoma* PCR-ELISA system's semi-quantitative potential.

The choice of a molecular method for diagnosing *Schistosoma* infection in endemic settings will depend on different factors, chiefly the available infrastructure and the cost-effectiveness. A control program may decide between performing multiple sample collections for the Kato-Katz examination or carrying out a single PCR-ELISA test, being the former less costly and the last more accurate. Considering only reagents, the cost of the PCR-ELISA system is roughly US$10 per fecal sample.

In conclusion, the *Schistosoma* PCR–ELISA system constitutes a precise tool for the diagnosis of *Schistosoma* infection, which may be particularly useful in low-prevalence settings and probably for post-treatment situations.
